# The age and cause decomposition of inequality in life expectancy between Iranian provinces: application of Arriaga method

**DOI:** 10.1186/s12889-022-13092-1

**Published:** 2022-04-15

**Authors:** Mehri Mehregan, Ardeshir Khosravi, Maryam Farhadian, Younes Mohammadi

**Affiliations:** 1 Department of Epidemiology, School of Public Health, Hamadan University of Medical Sciences, Hamadan, Iran; 2 Deputy for Public Health, Ministry of Health and Medical Education, Tehran, Iran; 3 Research Center for Health Sciences, Hamadan University of Medical Sciences, Hamadan, Iran; 4 Department of Biostatistics, School of Public Health, Hamadan University of Medical Sciences, Hamadan, Iran; 5 Social Determinants of Health Research Center, Hamadan University of Medical Sciences, Hamadan, Iran

**Keywords:** Inequality, Life expectancy, Mortality, Iran

## Abstract

**Background:**

This study aimed to decompose the age and cause inequality in life expectancy between two Iranian provinces with the highest and the lowest life expectancy using the Arriaga method.

**Methods:**

The required data was extracted from the death registration system (DRS) and statistical center of Iran. First, we calculated life expectancy at birth for 31 provinces of Iran using life tables, and subsequently, two provinces with the highest and the lowest life expectancy were determined. To decompose the age and cause share in the life expectancy gap between the two provinces, we used Arriaga's method.

**Results:**

Tehran with 80.09 years and Sistan and Baluchistan with 72.9 years had the highest and the lowest life expectancy among Iranian Provinces respectively. As a result, the life expectancy gap between Tehran and Sistan and Baluchistan was 7.19 years. Results of age decomposition showed that the highest share in the life expectancy gap attributed to the age group under one year (1.25 years). In terms of the cause of death, the highest percentage belonged to hypertensive diseases with a share of 1.77 years.

**Conclusions:**

There is a wide gap between two provinces with the highest and the lowest life expectancy. Age less than one year and hypertensive diseases were major factors in this inequality. Therefore, policy-makers should concentrate on improvement of survival in children and the reduction of hypertensive diseases to promote life expectancy in Sistan and Baluchistan.

## Background

Life expectancy (LE) at birth indicates the mortality pattern in all age groups, which estimates the average number of years that a newborn is expected to live, provided that the current pattern of mortality does not change [1]. Globally, this indicator is frequently used as a tool for measuring the progress and backwardness of countries. To such an extent that, any change in the life expectancy of population reflect improvements or reductions in mortality and living conditions [2–4]. However, LE in the different countries and regions is not identical and there is a disparity between and within regions. In the world, gap in LEbetween Highest and the lowest is approximately 34 years. Regarding the latest statistics disseminated by WHO, Japan with 84.3 years and Central African Republic with 50.7 years have the highest and the lowest LE in the world [5]. Moreover, this report shows that LE for Iran has been increased from 72.6 years in 2000 to 77.3 years in 2019. However, LE for all provinces of Iran is not identical, and like many regions, there is the disparity within county. Therefore, to reduce such disparity, exploring and identifying the factors affecting such disparity is required [6]. Many factors including socio-economic, racial, ethnical and geographical factors have role in inequality of LE [7–9]. The age and cause of death have the key role in making inequalities between communities that should be examined. Therefore, decomposing the age and cause of death factors contributing to inequality between communities may assist improving LEin communities with low LE [10–12]. To date, no study has examined the role of age and cause factors in inequality of LE between Iranian provinces, and our knowledge is limited. Therefore, in this study, we aimed to decompose the age and cause share in inequality of LE between Iranian provinces using Arriaga's method [13].

## Methods

### Data sources

In this study, we used two data sources; death registration and census. We obtained mortality data by age and cause for 31 provinces of Iran for 2017 from the Death Registration System (DRS) administered by the Iranian Ministry of Health and Medical Education (MOHME). This system collects the required data such as age, sex, residence, and cause of death for the deceased person. The death data occur in hospitals, health houses and health centers, forensic medicine bureau, authorized cemeteries, civil registration bureau and other probable sources are reported to MOHME. Subsequently, the staff of National Organization for Civil Registration (NOCR) of Iran classifies the leading cause of death by International Classification of Diseases (ICD) codes [14, 15].

Moreover, we extracted the number of the population from census data gathered by the Statistics Center of Iran.

### Adjusting death and population data

Prior to calculating LE for provinces, we calculated completeness of death registry using Synthetic Extinct Generation (SEG) method. This method yields percentage of death registration for DRS.

To overcome misclassification of cause of death, we re-distributed proportionally ill-defined codes of registered deaths based on ICD chapters. In this step, we obtained the proportions based on the distribution of the target ICD codes by age-groups, sex and province. Then, the ill‐defined causes of death were split proportionally over all causes of deaths registered based on ICD.

For evaluating the quality of population data, whipple’s index is used to measure age-heaping of census. Based on Whipple’s index, score of quality was 104.2, indicating high precision in age registration. Moreover, the studies confirmed that there is no under-registration or over-registration of population in census data, and therefore, coverage of census in Iran is not a concern [16].

### Constriction of LE

At first, we calculated LE at birth for 31 provinces of Iran using life tables [13]. Subsequently, regarding the retrieved results, we determined two provinces with the highest and the lowest life expectancy.

### Decomposing the LE gap

After identifying two provinces with the highest and the lowest life expectancy, we divided the absolute gap in LE between them into age and cause components using the Arriaga's method [2, 13, 17]. Arriaga distinguishes three different effects of mortality changes on life expectancy: a direct effect (DE), an indirect effect (IE), and an interaction effect (I). The direct effect is the change in the number of person-years lived within a particular age group (iLx) due to a mortality change in that age group. The indirect effect is the number of years added to (or removed from) a given LE because a mortality change within a specific age group produces a difference in the number of survivors at the end of that age interval. In the presence of unchanged mortality rates at older ages than the age group under consideration, the increase (or decrease) in the number of survivors at the end of the age interval results in increased (or decrease) in the number of years lived. Both the direct and indirect effects take into account mortality change in a specific age group, independent of the changes in other ages. Since mortality changes coincide in all ages, a small part of the change in LE is because the additional (or fewer) survivors (those responsible for the indirect effect) do not experience unchanged mortality at older ages. The impact resulting from combining the changed number of survivors at the end of the age interval and the lower (or higher) mortality rates at older ages is termed the interaction effect (I). Adding the direct, indirect, and interaction effect gives the total contribution of each age group to the change in life expectancy, or in other words, the decomposition of a difference in LE by age. In the second step, the contribution of each age group is further decomposed by cause of death, assuming that within each age- group, the contribution that a cause of death makes to the change in LE between time t and t + n is proportional to the contribution that this cause makes to the difference in the central mortality rate in that age group. The mathematical formulas describing this method are described below [13, 18].

### Decomposing by age

The total contribution of an age group to the LE gap (in years) is the sum of two mathematical terms, the first corresponding to a direct effect and the second to indirect and interaction effects, as follows:

$${}_{n}C_{x} = \left[ {\frac{{l_{x}^{Sistan} }}{{l_{0} }} \times \left( {\frac{{{}_{n}L_{x}^{Tehran} }}{{L_{x}^{Tehran} }} - \frac{{{}_{n}L_{x}^{Sistan} }}{{L_{x}^{Sistan} }}} \right)} \right] + \left[ {\frac{{T_{x + n}^{Tehran} }}{{l_{0} }} \times \left( {\frac{{l_{x}^{Sistan} }}{{l_{x}^{Tehran} }} - \frac{{l_{x + n}^{Sistan} }}{{l_{x + n}^{Sistan} }}} \right)} \right]$$

where nCx is the total contribution between ages x and x + n, lx is the number of individuals left alive at age x in a fictitious cohort, l0 is the cohort size at the start (commonly 100,000 in a life table), nLx is the number of person-years lived between ages x and x + n, and Tx+n is the total number of person-years lived above age x + n. The first term of Eq. 1 represents the direct effect of age, which consists of the number of years an age group adds to a LE differential, due to higher mortality in that specific age group in one population. To illustrate, if mortality between Sistan and Baluchistan and Tehran is equal at all ages except is higher in Sistan and Baluchistan for the age interval 40–44 years, LE in this province would be lowered by a direct effect of mortality of that age group, thereby widening the gap. However, there is also an indirect effect of the 40–44 years age group because the higher mortality in Sistan and Baluchistan leaves fewer survivors at age 45 years, affecting all later age groups in the life table [13, 18]. The second term of Eq. 1 represents this indirect effect. Furthermore, if mortality between Sistan and Baluchistan and Tehran differs at many ages, interaction effects are introduced, reflecting the continuously changing number of survivors and mortality rates of later age groups. The second term of Eq. 1 also captures these interaction effects. The last age group, however, has only a direct effect, because there is no later age group on which indirect or interaction effects can act [13, 18]. The next step is to sum the direct, indirect, and interaction effects to obtain the total contribution of an age group. The sum of contributions from all age groups should equal the total LE gap in years. It is also possible to calculate the ratio of the number of years contributed by an age group to the total gap, to estimate inequality on the relative scale [13, 17, 18].

### Decomposition by cause of death within an age group

The second step is to compute the contribution of causes of death to the LE gap. To do so, the total contribution of a given age group is further partitioned into the number of years contributed by each cause, as follows:

$${}_{n}C_{x}^{i} = {}_{n}C_{x} \times \left[ {\frac{{{}_{n}R_{x}^{i,Tehran} \times {}_{n}m_{x}^{Tehran} - {}_{n}R_{x}^{i,Sistan} \times {}_{n}m_{x}^{Sistan} }}{{{}_{n}m_{x}^{Tehran} \times {}_{n}m_{x}^{Sistan} }}} \right]$$

where nRxi is the proportion of deaths between ages x and x + n due to cause i, and nmx is the all-cause mortality rate between ages x and x + n. The total contribution of any given cause to the LE gap is obtained by summing cause-specific contributions across age groups. Similar to age, the sum of contributions from all causes should equal the total LE gap [13].

### Ethical consideration

Ethics Committee of Hamadan University of Medical Sciences endorses the study (IR.UMSHA.REC.1398.828).

## Results

**Fig. 1:**
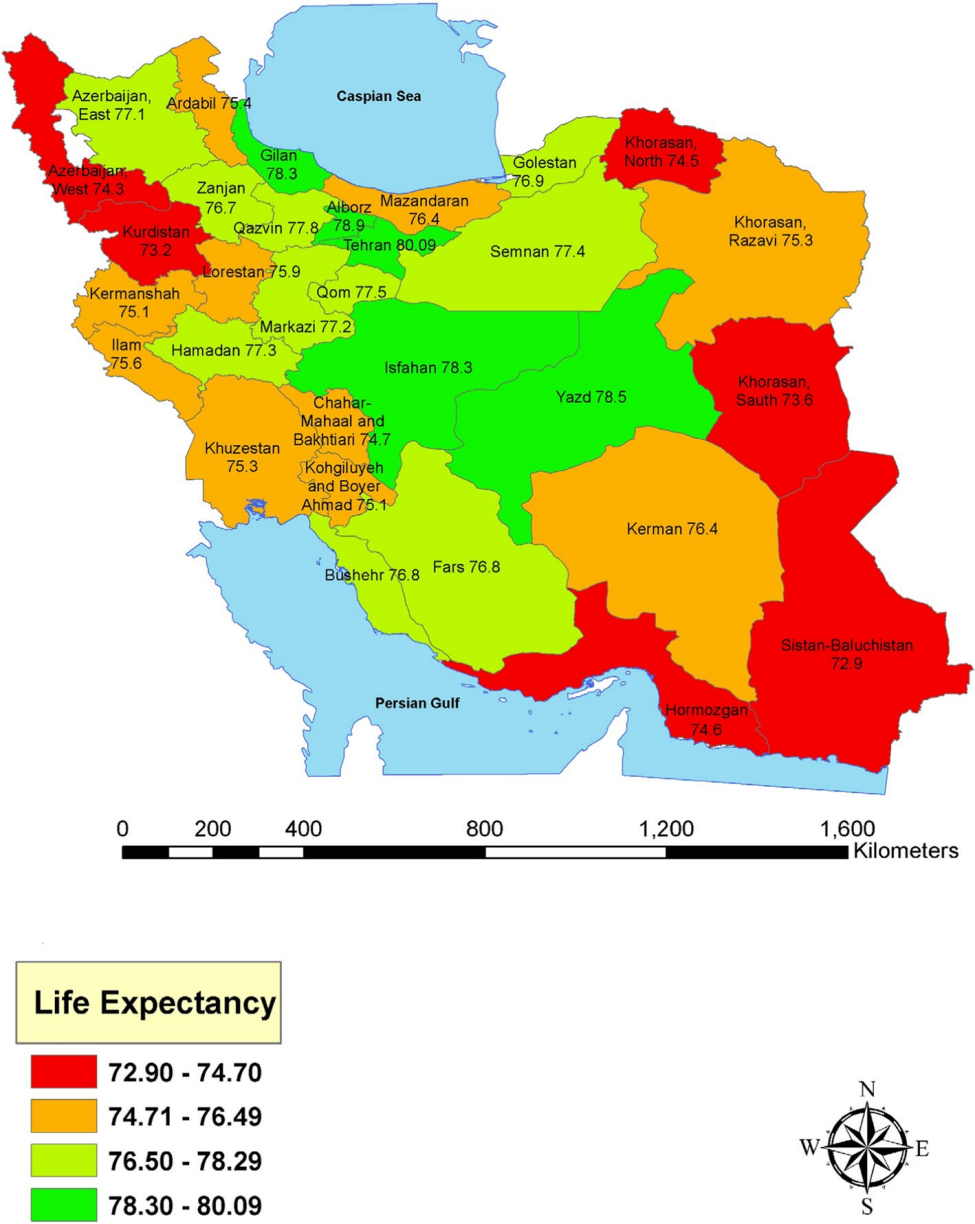
Life expectancy at provinces of Iran

Results for completeness of death registration in Iranian provinces is presented in Table 1. This table shows that the highest completeness belongs to Yazd, Qazvin and Isfahan with 99%, while the lowest completeness belongs to Golestan with 72%. Additionally, the table provides LE for 31 provinces. Tehran with 80.09 and Sistan and Baluchistan with 72.9 years had the highest and the lowest LE among the provinces of Iran. consequently, the gap in LE between Tehran and Sistan and Baluchistan was 7.19 years. Figure 1 demonstrates spatial situation of Iranian provinces along with LE for each province. Table 2 presents life table along with its values for Tehran province. Table 3 demonstrates life table along with its values for Sistan and Baluchistan province.

**Table 1:** Completeness of death registry and life expectancy for Iranian provinces

Province	Completeness of death registry (%)	Life Expectancy (year)
Azerbaijan, East	85	73.1
Azerbaijan, West	80	72.6
Ardabil	75	73
Isfahan	99	78.3
Alborz	95	78.6
Ilam	91	76.6
Bushehr	95	74.8
Tehran	98	80.09
Chahar Mahaal and Bakhtiari	82	77
Khorasan, Sauth	88	73.8
Khorasan, Razavi	92	75.3
Khorasan, North	88	74.9
Zanjan	91	76
Semnan	96	76.4
Sistan and Baluchistan	80	72.9
Fars	97	76.8
Qazvin	99	75.8
Qom	98	75.5
Kurdistan	90	74.2
Kerman	93	76.4
Kermanshah	98	75.1
Kohgiluyeh and Boyer-Ahmad	76	76.1
Golestan	72	74
Gilan	82	73.3
Lorestan	90	76.9
Mazandaran	90	76.4
Markazi	93	77.2
Hormozgan	86	75.6
Hamadan	94	75
Yazd	99	78.5
Khuzestan	97	74.3

**Table 2:**
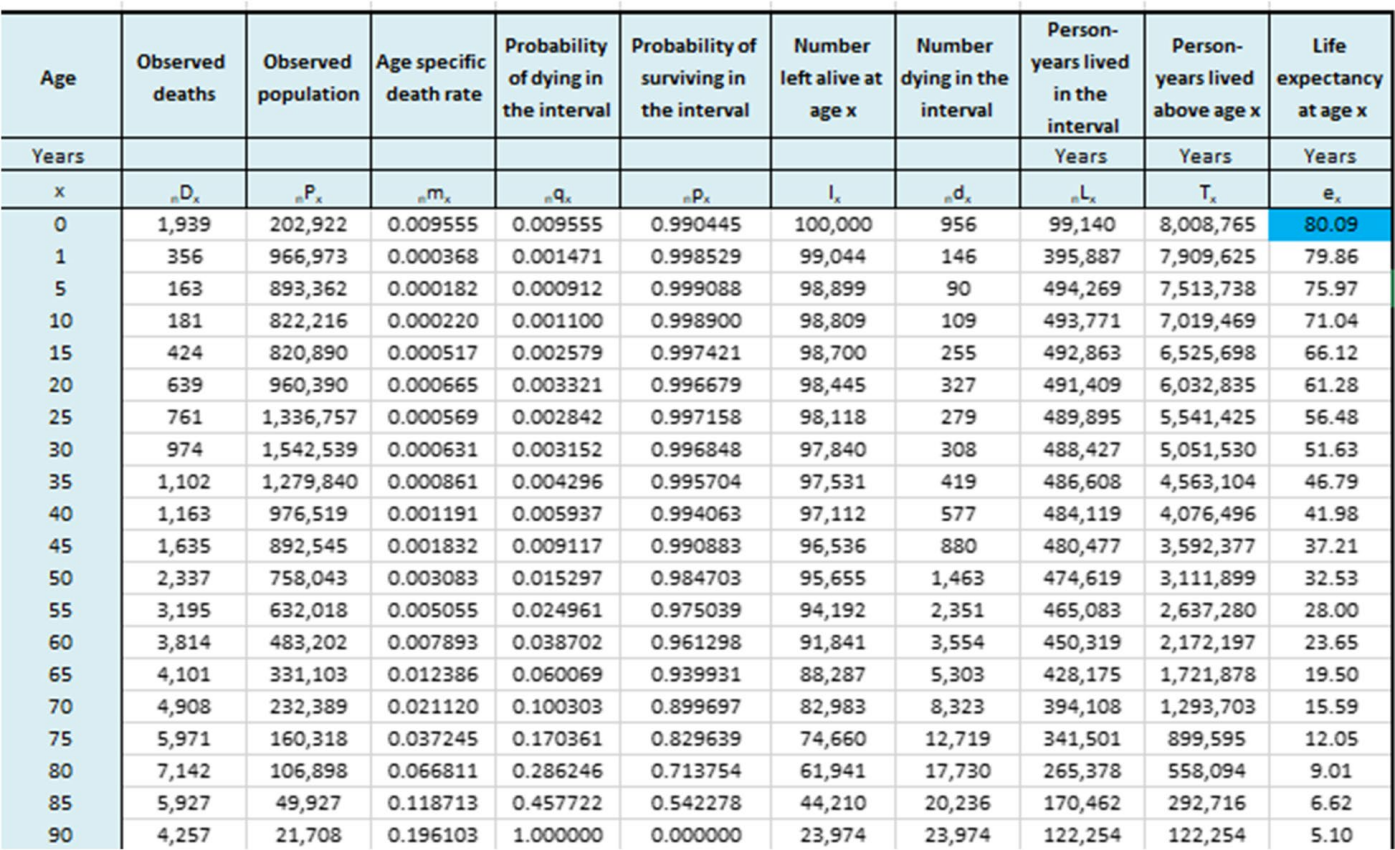
Life table with mortality rates for Tehran

**Table 3:**
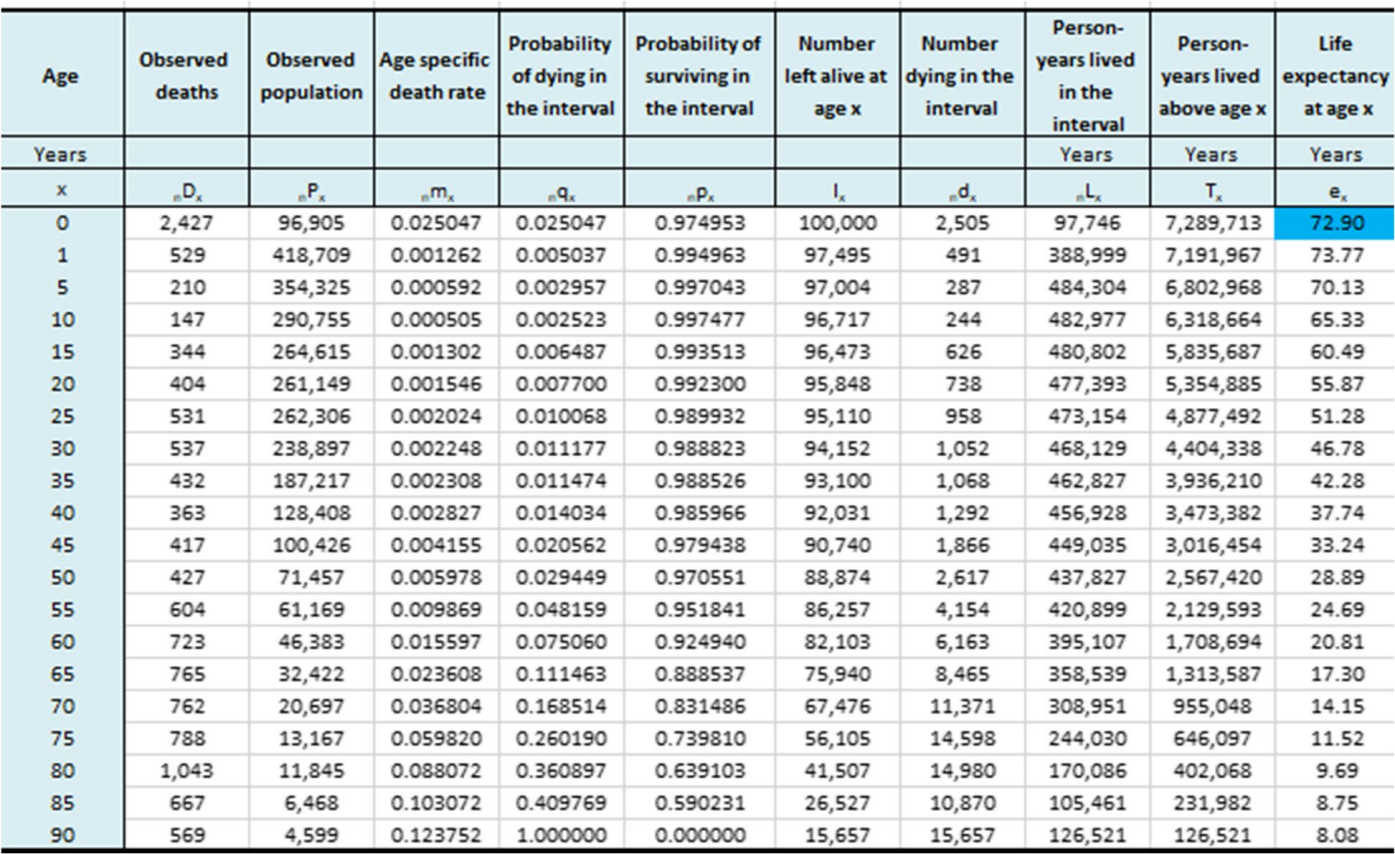
Life table with mortality rates for Sistan and Baluchistan

**Fig. 2:**
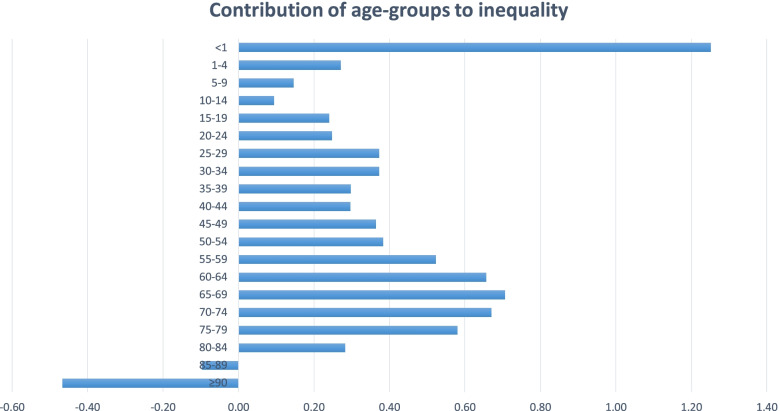
Contributions of age to the life expectancy gap

Figure 2 presents the result of age decomposition in LE between Tehran and Sistan and Baluchistan. The table shows that the highest share in the LE gap belongs to the age under one year (1.25), and the lowest percentage belongs to the age- group over 90 years (-0.47).

**Fig. 3:**
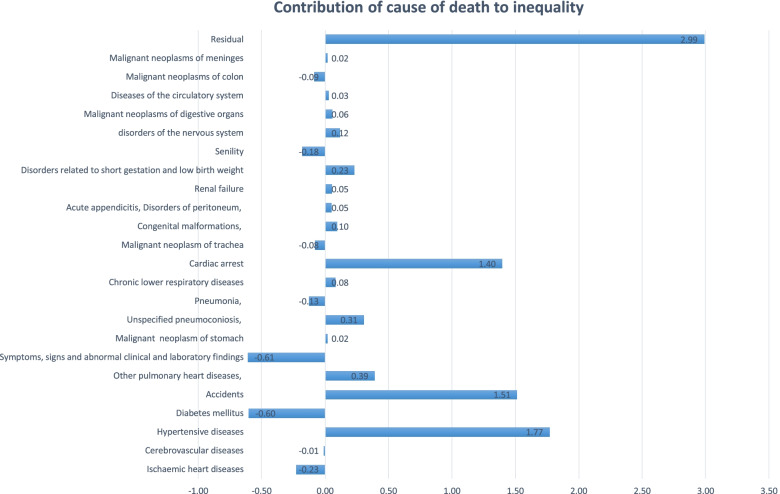
Contributions of cause of death to the life expectancy gap

Figure 3 demonstrates the decomposition of LE by cause of death. The result shows that the highest share belongs to hypertensive diseases with 1.77 years and the lowest share belongs to Diabetes mellitus with -0.60 years.

## Discussion

This is the first study that explores inequality in LE by age and causes of disease between Iranian provinces. This study found that there is a disparity in LE between provinces of Iran. However, Examining the characteristics of the provinces show that LE in the provinces significantly associated with socio-economic, demographic, medical and health factors. In general, Overall, the provinces with low LE have the common attributes. They have least income and literacy level, and rate of urbanization and access to medical and health services is low compared with the provinces with high LE [19–21]. Moreover, prevalence of malnutrition, child mortality in these provinces is high [22, 23]. Furthermore, the ethnic minorities comprise the dominant residents of the provinces [24]. Moreover, in this study, we found that The lowest LE belong to Sistan and Baluchistan and the highest LE belong to Tehran. Overall, this study demonstrated that the provinces with low LE have some common attributes. These provinces possess the low economic status, low literacy level, low nutritional level, ethnic and religious minorities. Sistan and Baluchistan province which has the lowest LE is a province located in south-eastern Iran bordering Afghanistan and Pakistan. Approximately, 4% of Iran reside in this province and Baluch is ethnic majority of people is Sistan and Baluchistan, which are of Sunni Muslims. On the other hand, Tehran is capital of Iran located in central Iran. Tehran Province with 18% of Iranian population, economically is the richest and most industrialized province of Iran, which produces approximately 29% of the GDP. Regarding census 2016, the least rate of literacy belongs to Sistan and Baluchistan with 76%, while Tehran with 92.9% has the highest rate of literacy. In terms of income, Tehran with 443,603 million Rials (national currency) and Sistan and Baluchistan with 233,729 million Rials had the highest and the lowest income respectively among the provinces. Furthermore, studies show that distribution of clinicians with diverse specialties and access to healthcare services is not homogenous around Iran. Central provinces, including Tehran, have most clinicians, while borderline provinces including Sistan and Baluchistan have least clinicians [19, 25].

Moreover, the decomposition results showed that age under one year had the highest contribution to the LE gap. In terms of the cause of death, decomposition analysis revealed that, hypertensive diseases were the dominant factor in inequality between Tehran and Sistan and Baluchistan. Therefore, to reduce this gap, policy-makers should focus on children's health through vaccination, nutrition promotion via Micronutrient supplements, such as vitamin A and zinc, improvement of integrated community case management (ICCM) of childhood illness, family planning, and maternal health. Furthermore, prevention of hypertensive diseases through keeping a healthy lifestyle may reduce the gap in life expectancy.

Several studies decomposed the age and cause inequality in LE between communities. After completing a decomposition analysis on the varying pattern of change in the sex differential in survival in the G7 (The Group of Seven) countries, which is an inter-governmental political forum include Canada, France, Germany, Italy, Japan, the United Kingdom, and the United States, it was found that the contributions of age- and cause-specific mortality to changes in LE vary from one country to another country. Cancer mortality, for example, reduce the LE disparity between males and females in Canada, the United States, England and Wales, and France, whereas it increases disparity in Germany, Italy, and Japan. In Japan, cause-specific mortality from accidents, violence, and suicide has widened the gap in LE between men and women [26]. Furthermore, a study published by Vesper et al. aimed at decomposing age- and cause-specific adult mortality contributions to the gender gap in LE from census and survey data in Zambia found that age- and cause-specific adult mortality positively contributed to the gender gap in LE at birth 50 percent of the time. The infectious and parasitic diseases, as well as accidents and injuries, were substantial cause-specific mortality positive contributors to the gender disparity in LE in the 15–59 age group. The age group 20–49, which is dominated by males had the major contribution to the gender LE disparity [27].

The LE discrepancy between Quebec and Canada with the same LE was found to be relatively small (0.1 years) when examining mortality disparities. Furthermore, gap analysis revealed that higher lung cancer mortality in Quebec was offset by the rest of Canada's higher cardiovascular mortality, and therefore, resulting in the same LE in both groups. Lung cancer was more effective in Quebec at a younger age, whereas cardiovascular mortality was more successful in older Canada [13].

Peters decompose LE for age and cause of death between residents of Inuit Nunangat and residents of the rest of Canada. He found that, in men, the major factor in LE gap between Inuit Nunangat and the rest of Canada was injury, particularly self-inflicted injury at ages 15 to 24. Moreover, he reported that, in women, the dominant contributor were malignant neoplasm and respiratory disease at ages 65 to 79 [28].

In this study, we used the Arriaga method to decompose inequality between Iranian provinces. This method has some advantages. Unlike directly standardized mortality rates, a standard population is not required. In addition, comparison of rate-based mortality studies is limited by the use of different standards. Besides, decomposition analysis uses only the data observed, accurately reducing the importance of all age groups and causes of death. Finally, these methods facilitate the identification of public health problems in the population by determining which specific age groups or causes of death have a significant impact on life expectancy [13]. the Results of this study may assist Iranian health policy-makers in declining inequality between the provinces. The policy-makers should improve the survival of children under five in Sistan and Baluchistan via escalation of childhood vaccination programs, control of malaria, improvement of nutrition, reduction of risk factors for hypertension diseases, and taking screening programs. In this study, we examine inequality between provinces, it is recommended exploring LE inequality between cities, between men and women in the future.

We had two limitations in this study. The data used in this study suffering two major problems; under-registration and misclassification of death. However, we calculated completeness of death registry using Synthetic Extinct Generation (SEG), and then the corrected number of death was used to calculate LE. This method assumes that all cases of death are under-registered homogeneously, the assumption which may not be correct. Moreover, the existence of ill-defined and garbage codes in the registered death may mislead the results. To overcome this problem, we proportionally re-distributed ill-defined and garbage records on all-causes or specific causes.

## Conclusion

This study provides the valuable information for Iranian Policy-makers to decline inequality in LE and improve the longevity of Iranian people. Age under one year and mortality from hypertensive diseases are the significant determinants of inequality in LE between Tehran and Sistan and Baluchistan. To reduce this gap, policy-makers should focus on children's health and prevention of hypertensive diseases.

## Data Availability

The data that support the findings of this study are available from the Iranian Ministry of Health and Medical Education (MOHME). However, due to policies of MOHME, restrictions apply to the availability of these data, and therefore, are not publicly available. To capture the data of this study, a written request should be delivered in Health Deputy of MOHME.

## Abbreviations

LE: Life Expectancy
DRS: Death Registration System
MOHME: Ministry of Health and Medical Education
DE: Direct effect
IE: Indirect effect
I: Interaction effect
ICCM: Integrated community case management
G7: The Group of Seven countries
SEG: Synthetic Extinct Generation
